# A foundational perspective of deontology and deontological codes through Paul Ricoeur´s “little ethics”

**DOI:** 10.1007/s11019-026-10362-2

**Published:** 2026-05-25

**Authors:** Nuno Ribeiro Ferreira, Pedro Bicho, Américo Pereira, Rui Nunes

**Affiliations:** 1 Bioethics Centre, Faculty of Medicine, University of Porto, Alameda Prof. Hernâni Monteiro, 4200-319 Porto, Portugal; 2 Internal Medicine Department, CUF Tejo Hospital, Avenida 24 de Julho, 171A, 1350-352 Lisbon, Portugal; 3 School of Arts and Humanities, University of Lisbon, Alameda da Universidade, 1600-214 Lisbon, Portugal; 4 Faculty of Human Sciences, Catholic University of Portugal (UCP), Palma de Cima Campus, Palma de Cima, 1649-023 Lisbon, Portugal

**Keywords:** Deontology, Teleology, Ethics, Medical judgment, Phronesis, Paul Ricoeur

## Abstract

Paul Ricoeur’s ethics comprises three distinct moments where it is possible to recognize the influences of Aristotle, Kant, Rawls and an original ricoeurian moment of practical wisdom, where the concept of phronesis is rehabilitated. When reflecting on medical practices, Ricoeur distinguishes, in continuity with his triadic ethics, three levels of judgment: the prudential and ethical level concerning the singularity of the clinical encounter that is essential for establishing a pact of trust, the formal and moral level where deontological codes emerge and the reflexive level where the two previous judgments become legitimated. Our aim with this paper is, through Paul Ricoeur´s ethics, to perspective deontology and deontological codes as a broader set of implicit philosophical and anthropological conceptions, rooted in the clinical encounter, that constitute a theoretical and foundational background for medical rules and norms.

## Introduction

The enormous development of medical sciences in the last century has altered medicine from an ancient art of caring to an ultramodern scientific discipline with a pretension of universality and based on criteria of objectivity and evidence. These characteristics are not isolated from the positivistic drift of human understanding about what science broadly is, and medicine in particular, as a scientific field applied to the person affected by illness. Somehow the intrinsic development of medicine and its dual nature as an art and a science propelled bioethical inquiry into new forms of reasoning and judgment due to the human implications of scientific exponential development and the consequent need for humanistic and holistic approaches to patients as persons (Nunes 2014). The reborn nature of medicine as an art of healing and the doctor‒patient relationship as the core for medical practice has also pushed medicine into the realm of philosophical inquiry, giving rise to new disciplines such as the philosophy of medicine through the work of doctors, bioethicists and philosophers such as Edmund Pellegrino (1981; 2001a) and David Thomasma (1990; Thomasma and Pellegrino, 1981). The universal pretension of the objectivity of medical sciences, and its implications for human life, was accompanied by the need for constant actualization and affirmation of medical deontological codes, as a set of rules, norms and expected behaviors from healthcare workers that care for other human beings. The medical deontological codes, ranging from the ancient Hippocratic Oath to modern versions worldwide, have not been immune to history. For example, the horrific actions of Nazi doctors have strongly encouraged the development of a modern code of ethics (Czech et al. 2023). The World Medical Association (WMA) was founded in 1948 after World War II, and the Geneva declaration (World Medical Association 2024a) was a document intended to prevent such terrible events from repeating (Parsa-Parsi, Gillon, and Wiesing 2024a). The latest 2022 revision of the International Code of Ethics by the WMA (World Medical Association, 2024b) is now facing new challenges imposed by globalization, multiculturalism, societal pluralism and diversity, as well as the reconciliation between deontology, virtue theory and consequentialism (Parsa-Parsi, Gillon and Wiesing 2024b). Interestingly, the original impulse of the Hippocratic Oath, which is the ultimate source of modern deontology, is much different from that of modern deontological codes worldwide. Hippocrates seems to have built a description of what constitutes a good doctor and good medicine, its ends, limits and applications (Stigall 2022), while the modern codes somehow respond to the cultural worldviews during a given period of history, changing themselves as the world and science evolve. Our objective with this paper is to develop a foundational perspective that enhances the anthropological and ontological background of deontology and deontological codes, whose rules, we defend, prefigure, not only formalistic determinations, but also aspects concerning ethics as *ethos*, that is, ethics as the core or habit of what is exactly human in the medical act. Thus, we understand that deontology and its normative codes go beyond the simple enunciation of rules and norms, and this beyond includes the unspoken or hidden aspect where an integral philosophy of the clinical encounter can be foreseen. The implicit meaning of the codes reinforces the ontological nature of medical ethics, being rooted in the clinical encounter between doctors and patients and reveals the ethical foundations on which they lay. We found Ricoeur´s triadic concept of ethics applied to the medical judgement very useful for our purpose of revisiting medical codes and show the untold philosophical background they tend to subsume and refund normativity in the clinical encounter. It is our belief that Ricoeur´s ethics unifies what bioethics sometimes tend to separate, namely, teleology, deontology and practical wisdom. Ricoeur is chosen because his ethics is structurally appliable to domains where principles are necessary but, if not anchored in the concrete setting, tend to be abstract and blind. Medicine is paradigmatic of that type of domain, as well as justice. The ricoeurian primacy of ethics as an aim over moral as a duty is also very pertinent for our aim of refunding deontology in the ethics of the doctor and patient relationship. Furthermore, Ricoeur was a philosopher that directly addressed medical audiences, through conferences and texts, showing how a philosophical thesis can have direct appliance into the scientific realm that, not so rarely, tends to marginalize theoretical and philosophical thinking. For our purpose, we follow Paul Ricoeur “little ethics”, as presented in the seventh, eighth and ninth studies of the work *Soi-même comme comme un autre* (1990b), and the application to the medical judgment that the French philosopher did in *Les trois niveaux du jugement médical* (Ricoeur 1996), later integrated in a volume called *Le Juste 2*. We also revisited the concepts of justice and institution through the dialogue that Ricoeur engaged with John Rawls, as presented in essays from *Le juste 1*, as well as Ricoeur´s rehabilitation of the Aristotle´s concept of phronesis. Additionally, we established a dialogue with Edmund Pellegrino and Thomasma´s philosophy of medicine and with Canguilhem´s vitalistic normativity, which also articulates ethics, norms and institutions at the praxeological and prudential level of the clinical encounter.

## From the singularity of the clinical encounter to deontology as a formal necessity

The doctor–patient relationship is regulated both by ethical determinations — within the category of the good — and by moral determinations — within the category of obligation. The determination of the action by predicates such as good and obligatory marks the relevance of *ought to be* as opposed to *being.* Both the doctor and the patient are active beings capable of action. The doctor’s action is intended to be based on precepts in the form of recommendations, advice and instructions capable of promoting good, the success of the cure and/or the relief of pain and suffering. These precepts simultaneously have a moral and technical nature in medical practice, which underlies good action.

According to Paul Ricoeur, the clinical and therapeutic relationships between doctors and patients arouse judgments at three different levels. The first is the prudential level in the setting of the clinical encounter where practical wisdom plays a central role. The second is the normative or deontological level where the norms and deontological codes emerge. The third is the reflexive level which encompasses the legitimacy of the two previous levels from an institutional perspective that includes the Rawls´s principles of justice (Ricoeur 1996). This triadic structure of medical judgment resembles, in a different order, the triadic conception of Ricoeur´s ethics(Nunes 2012) as “*the aim of a good life*,* with and for the others*,* in just institutions*”(Ricoeur 1990b, p. 202). In this context, it is worth emphasizing the distinction between ethics and morality in Ricoeur. The distinction is not etymological but conceptual: Ricoeur reserves the term “ethics” for the pursuit of a life lived under actions esteemed as good, and the term “morality” for the realm of the obligatory—that is, of norms, obligations, and prohibitions—characterized by a demand for universality and a coercive effect. Ethics is then related to Aristotle´s teleology and morality to a deontological Kantian heritage (Ricoeur 1990a). Concerning the relation between the two, Ricoeur defends the primacy of ethics over morality because the latter arises from a prior (ethical) intention. Despite its precedence, the ethical design must pass through the “sieve” of the moral norm, but when the norm generates conflicts, it is reasonable to return from morality to ethics, through practical wisdom (Ricoeur 1990b).

In the first level, the clinical encounter is the moment that best expresses the ultimate ends of medicine, constituting the unique situation of meeting between the doctor, endowed with knowledge and the patient, who expresses its circumstances of fragility and suffering and calls for a return to a previous state of health (Ricoeur 1996). Suffering is characterized by the diminution of the capacity to act, and here, Ricoeur approaches Spinosa´s idea of sadness as a primary affection that decreases that capacity and determines conatus, the universal and metaphysical striving to persevere in being and existence. Suffering, as Ricoeur explains, is felt as a threat to one’s integrity (Ricoeur 2013). The sufferer seems reduced to the condition of only being able to receive or can only give what is driven from the external and violent determinations of disease. The sufferer gives something that no longer comes from strength, but from his own weakness. In Spinosa´s terms, the patient is coerced, can only react and consequently is not free since he is no longer the sole determinant of his action (Espinosa 1992). The established bond between giving and receiving appears in the form of a pact of trust. This pact, at the level that Paul Ricoeur calls prudential, is the fundamental determination of medicine as a practice on the basis of “*social relations for which suffering is the fundamental motivation and the telos [purpose] is the hope of obtaining help and maybe be healed*”(1996, p. 22). At this first level of judgment, within the relationship between doctor and patient that is simultaneously an act and an encounter, the patient’s desire to be free from suffering is expressed by a personal narrative. Suffering, which should not be confused with pain, has an immediate existential character, as expressed in the sentence “*I suffer- I am*” (Ricoeur 2013, p. 17), with no ergo in between, as in the famous cartesian dictum *Cogito*,* ergo sum*. According to Paul Ricoeur, suffering is disruptive in two different axes: the axe of self-another and the axe of act-suffer. Both axes depict the sufferer as unique and suffering as a lived and irreplaceable experience, as well as solitary due to its uncommunicable feature. On the axe act-suffer, suffering decreases the ability to be and to act, which has implications for the possibility of action and discourse, in the capacity to self-narration and decreases the esteem of the self (Ricoeur 2013). The ricoeurian conception of suffering resonates in the clinical encounter, making doctor and patient relationship a true pact, built upon trust(Ricoeur 1996) and teleologically oriented toward the mitigation of suffering. In these circumstances, the doctor and patient are positioned at an existential level. From a primarily unequal encounter, “*on one side the one who knows*,* and knows how to do and on the other side the one who suffers*” (Ricoeur 1996, p. 23), the established pact of trust ends up reducing asymmetries, placing doctors and patients orbiting a common finality. Ricouer calls this pact an “*alliance*” (1996, p. 23). At this level of judgment, Ricoeur appeals for the need for prudence, heir of Aristotle’s idea of phronesis, also called practical wisdom. In book VI of *The Nichomachean ethics*, Aristotle describes five intellectual dispositions (hexeis) through which the parts of our soul capable of Logos can achieve truth: art, scientific knowledge, practical wisdom, philosophical wisdom and intuitive reason (Aristotle 2009, p.104). Practical wisdom or phronesis articulates a “ reasoned and true state of capacity to act with regard to human goods” (Aristotle 2009, p. 106), in the horizon of good life within the dominion of contingency, which encompasses variation and demands deliberation. Deliberation plays a central role in practical wisdom. In book III, Aristotle had already stated that we deliberate on “ the things that are brought about by our own efforts, but not always in the same way” (Aristotle 2009, p. 43). Deliberation is about the means, not the ends (Aristotle 2009, p. 44), in relation to which there is indetermination. Later in the book VI, Aristotle reenforces that deliberation requires a practical intelligence capable of recognize what is effectively good, in changeable and concrete circumstances, by practical apprehension of the singular and ultimate (Aristotle 2009, p. 113). This important feature of Aristotle´s ethics shows that practical reason does not operate through the same kind of reason that geometrical demonstration does. Instead, the wise man follows a model of judgment capable of attending reasons, recognize what is opportune and adjust the universal to the concretude of the case, in which practical truth is made operative. The rehabilitation of Aristotle’s phronesis for the clinical setting by Ricoeur, enhances that medicine not only deals with rules, norms and protocols but is directed to a particular person, which is vulnerable, suffers and deals with disease in his own unique way. No rules and principles can anticipate this existential singularity. According to Ricoeur, practical rationality always becomes effective in singular and concrete cases by conscientious deliberation that demands not only discursive reasoning, but also perceptive sensibility, correct evaluation of the appropriate mean to the good of the patient and interpretative competence (Ricoeur 1997). Through phronesis we navigate between generality and particularity (Boudreau et al. 2024), and *phronimos*, the wise and prudent man, defines both the case and the rule (Ricoeur 1990b) in a broader vision that integrates virtue, intuition, experience and general rules. Additionally, he is able to deliberate about the right means to accomplish the ends of the action and the circumstances that define the object of action (Pereira 2012). In the practical realm of clinical encounter, the place of suffering that we previously defined now meets the solicitude of the doctor, not fundamentally linked to the fulfillment of a duty or norm, but with what Ricoeur called a “*compliant spontaneity*”(1990b, p. 222) or a genuine will to help based of practical wisdom.

The question, then, is why there is a need for normativity? Would not the nature of the relationship established in the clinical encounter—grounded in trust and governed by the virtue of phronesis, raised to the level of a pact—already serve as the guarantor of the alliance’s reliability? According to Ricoeur, the pact is fragile and constantly threatened by distrust and suspicion (1996). On the doctor’s side, there are dangers such as decay in the objectification of the human body as well as the potential conflict between public and private health; on the patient’s side, the pact can be weakened by the fear of violence and abuse of power and the doctor’s inability to respond to their needs (Ricoeur 1996). Because of the frailty of the pact, which is constantly threatened by external and internal determinants, proceeding to a normative and deontological level of judgment is mandatory. We now orbit a different level of judgment, the deontological level. It is interesting to notice that, apart from the pretention to be universal and rational, deontology is somehow intrinsically connected with the act itself. For example, the dissymmetric nature of the clinical encounter, so much exposed to the threats of violence and abuse of power, immediately appeals for the need to treat all persons as ends in themselves, as expressed in Kant´s second categorical imperative. Besides its formalistic aspect, deontology is not only externally imposed, as the codes seem to be, but its impulse is driven directly from doctor and patient relationship, therefore being also constitutive. Thus, the functions of the deontological level of judgment are to universalize the precepts of the care pact, leading to the establishment of imperatives, and to arbitrate the conflicts that arise owing to the frailty of the singular relationship (Ricoeur 1996). Although conflicts seem to be, in some way, hidden in deontological codes, normativity enunciates certain general and consensual principles, which can offer a solution to conflicts that arise at the level of prudential judgment. Many of the previously mentioned conflicts arise from the confrontation between the development of medicine and the biological sciences that support it. The patient and his body is, at the same time, the object for biomedical research and concern for clinical care. Experimental research on human beings frequently involves excessive objectification of the body, especially in double-blind scientific studies, in which neither experimental subjects nor researchers know in detail all the particularities of the interventions. From the tension raised by the consideration of the body as the scientific object and the person as a human being with dignity, which can also be threatened, conflict may appear, and deontology itself emanates as a pacifier of those conflicts. In this formal level of rules and norms, informed consent is an instrument that not only affirms autonomy within the state of vulnerability (Portocarrero 2013) but also elevates the patient to the category of partner, somehow reducing the asymmetries of the first level and ensuring the preservation of dignity. Ricoeur will tell us that there are three pillars of normativity: medical secrecy, the right to the truth and informed consent (1996). These pillars constitute the normative stronghold in the face of conflict arising from relational singularity and from the paradoxical nature of medicine, as an art and a science. These paradoxes are presented as follows: “first, the human person is not a thing and yet his body is a part of the observable nature; second, the person is not a commodity, nor is medicine a trade,” but medicine necessarily implies a cost, that means having a price for users and society; last paradox, which overlaps the two previous ones: “suffering is private, but health is public” (Ricoeur 1996, p. 29–30). Due to the paradoxical characteristic that clinical relationships can assume, ethics is enriched by submission to the deontological norm, and in certain cases, there is a need to resort to the law in the arbitration of conflicts (Ricoeur 2001). However, we maintain that normativity and biolaw serve primarily to resolve conflicts and dilemmas, rather than to define the essence of medical action, which is grounded more fundamentally in the personal, existential, and virtue-based encounter between physician and patient. We follow Ricoeur when he clearly affirms the “primacy of ethics over morals” (1990b, p. 200-1). The necessity for ethics to pass through the critical examination of normativity and the formal legitimacy of norms is not free of new conflicts, as norms and rules can contradict each other. These second-level conflicts may be attenuated by returning to the level of praxis, where practical wisdom and deliberation are useful tools to solve some issues at the deontological level (Ricoeur 1990b). The ethical essence resides in the «aim of a good life» as an intention to be good (Portocarrero 2011), patent at the prudential level that imposes itself over the moral level that proceeds by establishing the rules to be good (Simms 2003). Thus, the meaningful value, or the properly called ethical level, is more related to the intention and desire to be good in practical situations than to the fulfillment of a particular rule or norm (Potvin 2010).

## The clinical anchor of deontology: from Aristotelian virtues to Kantian imperatives

The intention to be good as an aim or, in Ricoeur’s own words, a “visée” (1990, p. 202) is typically an Aristotelian feature, with the intention being followed by the right and appropriate means for prosecuting what Aristotle called “eudaimonia” (Ricoeur 1990a). Ricoeur’s appeal for the practical horizon of Aristotle´s ethics, which is an ethic of action and practical wisdom that is teleologically oriented, conceives good as an end in itself, achieved by exercising virtues applied to deliberation and action (Anagnostopoulos 2009). After the “visée”, the reference to the others and to institutions ruled by justice, in Ricoeur’s triadic definition of ethics as “the aim of a good life, with and for the others in just institutions” (Ricoeur 1990b, p. 202), links the self in his ability to esteem of his own, to the other human being as an equal, and then to a third entity, person or community, bearer of rights under the legal, social and political plan (Ricoeur 2021).

The Aristotelian and Kantian features are subsumed in Ricoeur´s moment of practical wisdom, which concerns the singular moment of the care pact. Instead of opposing Aristotle’s ethics based on teleology and eudemonistic tradition and Kant´s deontology, Ricoeur shows that those traditions call for each other, and the place of the wish, in Aristotle ethics, has some correspondence to the good will in Kant’s theory, a will whose autonomy is driven from self-legislation, assuming itself as practical reason (Denis 2010). If ethics is essentially intentional, morality is a way to make that intention effective in the face of forces that constitute a threat, such as violence (de Brito 2008).

In some sense, we can affirm that Aristotle’s virtue theory touches Kant’s idea of maxim. Kant’s ethics is a deontological ethics, an ethics of duty, in which the consequence of action is not the determinant of moral value but rather the action of a genuine “good will” (1998, p. 7). Kant draws attention to the superiority of duty as a criterion for guiding action, a duty that, far from being externally imposed, is the choice of reason itself. It therefore constitutes an absolute, *à priori* and transcendental substrate. Reason provides itself with laws called imperatives, and the categorical imperative—acting according to a maxim that can be considered a universal law—is objective, universal and logical. The apparent opposition between teleology and deontology, interiority and exteriority, can also be seen as an ontological approximation. If Aristotelian virtues point to the ontological center of the human being, actualized in praxis through practical wisdom and action, Kantian deontology will also find the supreme source of morality in the autonomy of the will. The autonomy of the will lies in fulfilling a maxim that is primarily rational, also pointing to an internal and human *locus*, simultaneously individual and universal, instead of constituting a mere prescriptive deontology by external imposition.

Ricoeur´s effort to reconcile both deontological and teleological ethics has brought into discussion the importance of both prudential and formal levels of judgment that have different features and intentions. Our epistemological position follows the one Ricoeur has proposed as the ultimate meaning of ethics, which lies in the particular circumstances of the clinical relationship where medicine´s nature as a practical art and science of healing comes into existence.

The perspective of a good life, as an ethical intention, is assumed as a vow, appealing to the moment of praxis that is realized in the capacity for intentional action and initiative. At the level of the relationship between two human beings, doctor and patient, in a fundamental praxeological sharing background, the esteem of the self, the starting point of Ricoeur´s ethical aim, unfolds into reciprocity. At this level, we do not consider normativity, or morality as Ricoeur understands it, but we are exactly at the primordial ethical level in which the norm will anchor itself, with the objective of universalization. Additionally, in clinical encounters, the pact of trust and the promise to keep that pact constitute the cornerstone of medical ethics. When elevated to the formal plan, this pact assumes the characteristics of a contract on the basis of the obligation of confidentiality and assumes the negative form, for example, in the interdiction to break medical secrecy (Ricoeur 1996). The universal character of the norm binds every doctor and every patient, and what was previously a confidence pact between two human beings now becomes a formal interdiction, universalized under the rules of normativity.

Although the movement from the prudential level towards normativity and formalism is intended to strengthen the doctor and patient therapeutic pact on the basis of trust, the rules of deontology are not free from new disputes, when the ethical principles conflict with each other. These second-order conflicts are evident, for example, in the tension between autonomy and beneficence principles or between confidentiality and public health issues. Although sometimes deontology must appeal to biolaw to resolve some conflicts, we follow Ricoeur when he proposes a solution by returning to a prudential level, endorsed by practical wisdom. Medical dilemmas and complex bioethical questions are present in everyday practice. Because some of the conflicts arise in a broader context that involves philosophical questions, particular values and worldviews, as well as existential questions, the solution must appeal to the basic features of medicine as an art and science oriented toward the healing and caring of those who appeal for help. We need to return to the unique situation of the clinical encounter, at an existential level where two human beings and two worlds meet and where medicine appears as a personal and practical relationship teleologically oriented toward patient wellbeing. As diversity is one of the main characteristics of clinical relationships, the solution for conflicts must be established within the clinical encounter rather than in the generality of rules and norms that tend to underestimate the variability and uniqueness of the medical context. The use of practical wisdom or phronesis, which directly appeals for initial ethical intuition, as guidance for action and problem solving, constitutes one of Ricoeur´s most interesting ethical proposals. The norms of the deontological codes claim their strength and legitimacy, not by the formal and moral imposition of rules, but by anchoring themselves in the fundamental aspect of medicine as an action oriented toward the good of a particular patient, by the solicitude of a particular doctor, in the personal relationship they establish.

## The institutional underpinning of ethical practice

The third part of Ricoeur’s little ethics refers to just institutions. The “aim of a good life” is not limited to interpersonal relations and virtues but extends into institution as the structure of the “living together of an historical community” (Ricoeur 1990b, p. 227). The ricoeurian triadic ethics concerning the aim, the others and the institutions is not a discontinuous path but an integrated one, and institutions are places of mediation where the tensions between the constraining norms and the singularity of the cases appear. In other words, institutions are the locus of human action. If the “aim of a good life with and for others” appeals for a proper ethical intention and desire to a good life from a dialogic structure of ipseity (I-You), institutions, as ruled by the virtue of justice, refers to the others as “anyone” or “to which is own” (Ricoeur 2000; p. XIII). There is an objective distance between justice/institutions, and interpersonal relations/virtues, or, in other words, justice and institutions seem to be more related to Kant´s deontology (what is just) than to Aristole´s teleology (what is good). This is also evident, as Ricoeur tells us in the third essay from *Le Juste I*, in the deontological and contractualist drift of John Rawls conception of justice as fairness, applied to the institutional and social aspect of human life. If, as Rawls defends, is possible to define principles of justice by a contractualistic procedure, following an imagined scenario famously known as the veil of ignorance that constitutes what Rawls calls «justice as fairness»(Rawls 1999, p. 10), the question is if it is possible to configure justice merely as the abstract result of this kind of procedure without attending the previous or foundational notion of common good. Ricoeur asks what kind of justice can be thought of as merely processual and apparently disconnected from any assumption of what is good, at the original position where the principles of justice would be chosen? If justice is, in fact, “the main virtue of social institutions, as the truth is of systems of thought” (Rawls 1999, p. 3), then justice is not simply a virtue among virtues, but the foundational virtue in which all others are possible that can´t be achieved without considering the aim or desire of human beings for a good life. The deontological focus is not omitted by Rawls, nor criticized by Ricoeur. What Ricoeur asks is whenever a justification for the procedural theory of justice exists that is nor procedural or deontological, but built upon the *ethos* as the aim teleologically oriented towards what is good. Here Ricoeur defends that there is a sense of justice rooted in the golden rule, or in the good as an intention teleologically oriented, that is presupposed in Rawls ´procedural justification of the principles of justice (Ricoeur 2000, p. 37). In this intention we find the bridge between the procedural notion of justice and the ethical principle, which is presupposed. The ricouerian bridge seems to reconfigure the just between the good and the legal (Ricoeur 1991), the first marking the extension of interpersonal relations to institutions and the second giving the law its coherence and right to coercion (Ricoeur 1990b). This reconfiguration allows mediation between institutions and persons that can be useful for the bioethical dilemmas concerning the constraints of health systems, the deontological codes and the doctor and patient relationship.

Health institutions are places of mediation where the three aspects of Ricoeurian perspective, namely the ethical, the deontological and the legal becomes operative. It starts with the ethical demand of a doctor who responds to a patient who calls for help, but goes beyond this bond because it takes place in an institution where considerations on the justice of distribution of resources can assume a procedural appearance. In a Rawlsian framework, hospitals apply justice through publicly shareable rules and procedures that prevent arbitrariness, allocate scarce resources, stablish criteria that sustain economic stability and proportionality. This procedural institutional justice, if left alone, can degrade the relationship between a patient in suffering and a caring doctor in an administered interaction. A clinical decision can be seen solely as the result of what the protocol says or an algorithmic decision considered good, only and precisely because of that conformity. The same happens by the blind application of norms and principles that rule the medical practice. The problem with this self-sufficient deontology is that it is necessary to prevent arbitrariness with transparent criteria, but not sufficient to ensure the ethical quality of the medical act. And, as Ricoeur tells us, a procedural theory of justice seems to left a remnant or «a résidu»(Ricoeur 1990b, p. 265) that demands the return to a teleological point of view, not to deny the necessity of formalistic procedures, but to build them upon an ethical claim that people genuinely want and desire. These remnants of procedural formalism in health institutions and in deontological codes, or what is left unsaid, are the zones where rule application and deontological principles demand the need of constant interpretation and judgement. Also, they urge to consider patients, not only as claimants of goods provided and distributed by institutions, but as authors of their own life narratives that determine what vulnerability, respect, justice, harm, benefit, good and evil means in their concrete situation. The risk of institutional reification by a checklist formalism or an abstract deontology can turn patients into passive receivers, impersonal clinical cases or legal objects of consent or refusal, and doctors into functionaries, rather than agents of prudential judgment. The institution, necessary for fairness, can degrade care ethics by dislocating the Ricoeurian “aim of a good life with and for others” for metrics of productivity and units of throughput, creating constrains that can corrupt solicitude and patient´s narrative. This is evident, for example, in time-limited appointments, in early discharges by the urge for available beds and in informed consent, so often reduced to a mere signature or a guarantee of institutional non-liability. Also, by dislocating decisions to committees, guidelines, and triage and allocation protocols, institutions can contribute to moral unaccountability, precisely where Ricoeur urges for the need for ethical justification and fairness. Reification risk can be overcome by the mediation of a just healthcare institution, by the precepts of Rawls, that require procedural transparency, but also accountability and public contestability, interpretative and contextual judgment and deliberation. Even Rawls refers to the necessary “reflective equilibrium” between conviction and social contract theory (Rawls 1999, p. 18). Norms and rules are indispensable, but they do not eliminate the need for practical wisdom that prevents the errors of both an excessive moral formalism and a romanticized arbitrary situationism.

Not only little ethics but also Ricoeur´s conceptions on narrative identity and hermeneutics has important implications on the way doctor approach their patients, value their life stories and their experience of being ill, therefore, changing institutional care or patient centered care. An interpretative and dialogical character in clinical setting is useful to avoid paternalism by valuing narrative and involving patients in the decisions concerning their care (Russo 2021). Also, Dorey has shown, in a very concrete situation, that the ultracomplex healthcare information management that include electronic records, genomic databases, biobanks and big data analytics may be threatened by breaches of confidentiality, asymmetric relations between doctor and patients regarding data, as well as potential power abuse and vulnerability to non-consensual uses of data. The author, then approaches healthcare information systems through a narrative and interpretative process, by using an ethical matrix based on Ricoeur´s domains of the self, others and institutions, in which patients became a participants capable to understand and decide what data they share, the others appear in a relation of reciprocity, benevolence and trustworthiness, and institutions ensure equitable participation, fair dissemination of benefits and data transparency (Mouton Dorey 2016).

Most ethical dilemmas and current bioethical debates appear in the tensional transit between solicitude, respect of norms and institutional justice. For example, Ricoeur shows, in the essay Autonomy and vulnerability, through the lens of judicial judgment, that the concept of autonomy, far from being a self-sufficient attribute to the subject, must be considered in a structural correlation with the threatens to the capacity to “speak”, “act”, “narrate” and being an agent of imputation that constitutes vulnerability (Ricoeur 2007, p. 73). Hettema will argue that care ethics may benefit from the overcoming of false opposition between autonomy and vulnerability by considering patients vulnerable but still capable beings, whose autonomy is affirmed within fragility and by situating personal narrative as an essential feature of autonomy. Also, Hettema argues for the need to dislocate abstract individualistic autonomy to a relational anthropology, in which autonomy is dependent on healthcare’s and patient´s capacities to speak, to hear and be heard, to recognize and being recognized (Hettema 2014). As care ethics is not solely oriented to patients as receivers of care (van Nistelrooij et al. 2014), the Ricoeurian hermeneutics of the self and the notion of fragility has been useful in recontextualizing care also as an integrating part of self-identify of caregivers. Because the self already incorporates otherness, caring for others is less related to choice or a loving and altruistic surplus than to a practice in which oneself “gives”, at some expense of self-sacrifice, but it’s constituted within that gift (van Nistelrooy 2014).

## Beyond normativity and formalism: the untold of deontological codes

Paul Ricouer assigns an important function to deontological judgment, although he places the proper ethical significance at the prudential level (1996), where caring for suffering is the foundational feature of medical action (2001). The importance given to the unique situation of the clinical encounter is also defended by Edmund Pellegrino, who places the nucleus of ethical meaning in this praxeological realm of the healing relationship that defines medicine´s nature. (Pellegrino 2001b). As we have seen before, deontological judgments are intertwined with prudential judgments. One of their essential functions is to universalize maxims contributing to the resolution of conflicts that arise at the first level of medical judgment. While the first level appeals to the excellence of virtue in practice, the second level is more formal, logical and autonomous. The need for universality and objectivity, still in a Kantian sense of seeking a universal foundation for morality, tends to value the rule more than the case. Because of their intention towards objectivity, deontological codes inevitably end up expressing consensual understandings that support the rules. Codes were written in a specific time and place; therefore, the conceptual background that forms the framework of the norms and rules is not disconnected from the philosophical, cultural and political conceptions of the times. Thus, for example, the enlightenment spirit and rationalism ended up increasing the value of patient autonomy, which, at earlier times, was subdued to medical paternalism and the authority of masters’ opinions. The Judeo-Christian conception of personhood, in its individuality, autonomy and irreplaceability, also changed the relationship between doctors and patients, both recognized as beings of freedom, choice and project, ideas very dear to existentialist philosophies, both Christian and non-Christian. If medical codes pretend to constitute, in their reflexive function, a set of rules and practices for medicine, one fundamental question arises: in what background can one find legitimacy for the codes in their coding task? There is, as Ricoeur tells us, a historical set of moral ideas underlying and not clearly stated in the norms of the codes governing the practice of medicine (1996) that provide ethical support for the codes, in a similar way of Rawl´s reflective equilibrium. The norms of regulation inevitably reflect various moral sources that lead to philosophical conflicts, where human reflection is broadly open to issues concerning life and death, good and evil, suffering and happiness, and health and disease. The last topic on health and disease, and the limits imposed on the conception of normal and pathological, has been explored by Georges Canguilhem. This author argues that the pathological is not a mere measurable and quantitative deviation from the physiological. Life is inherently normative because living organisms do not only adapt to environment, but they actively institute their own physiological milieu, establishing new norms and values in disease. Disease is therefore “not the absence of norms but the presence of other norms” (Canguilhem 2008, p. 131). It is by the determinations that disease have in life, by diminishing patients “from an active and comfortable participation, generative of confidence and assurance, in the kind of life previously belonging to it and still permitted to others” (Canguilhem 2008, p. 131–132) that disease is brought back from the normative aspects of statistics to the existential level of a lived experience and clinical relationship. This movement is dear to Ricoeur´s foundation of deontology, and ultimate justification of the rules of respect, by moving back to the prudential level of judgment. In the horizon of concrete life where normativity and interpretation are used for “targeted respect” that is owed to patients who live disease and disability, this respect is determined by the comprehension and interpretation of the proper difference that constitutes the pathological state (Ricoeur 2007, p. 187–197), therefore extending Canguilhem´s vital normativity to the hermeneutical and ethical plan.

Justice, and the requirement to appeal to law to resolve some conflicts in medical scenarios, are also socially and culturally embedded and face the same question of legitimacy (Ricoeur 2001). Deontology, and we follow Ricoeur in this idea, ends up being rooted in philosophical ontology and anthropology, about a certain sense of humanity, which inevitably does not escape the versatility of the history of philosophical ideas. Therefore, far from the normative intention, the philosophical basis of norms constitutes the ground that Paul Ricoeur imports from John Rawls’ theory of justice as built upon consensual agreements and reasonable disagreements (1996). Deontology functions as a sieve for judgments at the prudential level, enhancing normative morality with a sense, a configuration, and a teleology that is not explicit in the codes. The third reflexive judgment consider prudential relationships and deontology inseparable and necessary for ethical realization. The philosophical support of deontological codes can and should be rooted in the praxiological realm of the *vis-a-vis* encounter between doctors and patients, for which suffering, hope of health and well-being and doctor solicitude constitute the *primum movens* of medical action. This philosophical background of normativity is clearly expressed in the ancient Hippocratic oath and still resonates with modern bioethical codes. In previous works, David Thomasma and Edmund Pellegrino noted that there are principles of morality arising from medicine itself as the science and art of healing and caring and not from external moral codes (2001;1997;1990). Additionally, the philosophy of medicine, as a field of knowledge establishing itself as a theory of medicine, may well provide the foundational background for medical ethics. (Pellegrino 1998; Thomasma 1980; Thomasma and Pellegrino, 1981). What we previously affirm about the untold or not explicit in medical codes, following Ricoeur, is precisely the foundational background for bioethics that the philosophy of medicine, maximizing the end and telos of the medical act, can also provide. Medical deontological codes worldwide are instruments for the professionalization and institutionalization of medicine (Jotterand 2005), are culturally and socially embedded and are therefore continuously submitted to change. The set of rules and norms they constitute will be ethically empty and permanently put in doubt unless they can be rooted on an anthropological and teleological level, defended by both Ricoeur and Georges Canguilhem, who affirms that “human biology and medicine are, and always have been, necessary parts of an “anthropology.” But we also hold that there is no anthropology that does not presuppose a morality, such that the concept of the “normal,” when considered within the human order, always remains a normative concept of properly philosophical scope” (Canguilhem 2008, p. 133). Placing the philosophical background of the codes in the heart of medicine´s essence and nature expressed in the clinical encounter may provide a solid background for normativity and prevent the artificial and harmful disconnection between deontology and the teleological and ethical aspects of medical practice.

## Conclusion

Paul Ricoeur created an original interpretation of the relationship between doctors and patients in therapeutic and clinical practice. From an intimate moment of interpersonal communion between a patient who appeals and a doctor who responds to that appeal, where a fundamental ethical pact of trust and treatment is built, much like Pellegrino and Thomasma’s conception of the telos of medicine, we move to a normative and formal level that allows the resolution of conflicts held at a prudential level and allows for the universalization and rationalization of prudential judgments. At a normative level, where ethical codes of conduct are established, norms serve as a bridge between deontological and prudential levels and ultimately enrich the clinical relationship itself. The tendency toward the universalization and objectivity of deontological ethical norms, we defend, should not be separated from a teleological perspective of the prudential level, which is the heir of the Aristotelian notion of practical wisdom or phronesis. The deontology to which Ricoeur appeals, always subsumed in ethics and expressed in general and rational norms, should not be devoid of purpose and meaning; the rules of the codes do not stand alone but rather emerge interconnected with an ultimately profound ontological background that is implicit. The third reflexive level of judgment appeals to the prudential-deontological-reflexive set, where notions that touch all bioethical inquiry are at play, such as the conceptions of life and death, health and disease and the ontology of human suffering, which result from philosophical debate held in a certain historical–cultural context. This philosophical background, which expresses the ultimate goal of medicine and its essence, patents in the clinical relationship, is where deontology must be anchored, overtaking the old opposition between teleology and deontology. Ricoeur has brought to medical ethics a true integral anthropology and ontology that, although not clearly stated and expressed in codes, is what allows the achievement of the good, the just and the legal, in the dramatic and eventually tragic aspects of human and medical action.
